# Enhanced Polysaccharide Binding and Activity on Linear β-Glucans through Addition of Carbohydrate-Binding Modules to Either Terminus of a Glucooligosaccharide Oxidase

**DOI:** 10.1371/journal.pone.0125398

**Published:** 2015-05-01

**Authors:** Maryam Foumani, Thu V. Vuong, Benjamin MacCormick, Emma R. Master

**Affiliations:** Department of Chemical Engineering and Applied Chemistry, University of Toronto, 200 College Street, Toronto, Ontario, M5S 3E5, Canada; INRA, FRANCE

## Abstract

The gluco-oligosaccharide oxidase from *Sarocladium strictum* CBS 346.70 (GOOX) is a single domain flavoenzyme that favourably oxidizes gluco- and xylo- oligosaccharides. In the present study, GOOX was shown to also oxidize plant polysaccharides, including cellulose, glucomannan, β-(1→3,1→4)-glucan, and xyloglucan, albeit to a lesser extent than oligomeric substrates. To improve GOOX activity on polymeric substrates, three carbohydrate binding modules (CBMs) from *Clostridium thermocellum*, namely *Ct*CBM3 (type A), *Ct*CBM11 (type B), and C*t*CBM44 (type B), were separately appended to the amino and carboxy termini of the enzyme, generating six fusion proteins. With the exception of GOOX-*Ct*CBM3 and GOOX-*Ct*CBM44, fusion of the selected CBMs increased the catalytic activity of the enzyme (*k*cat) on cellotetraose by up to 50%. All CBM fusions selectively enhanced GOOX binding to soluble and insoluble polysaccharides, and the immobilized enzyme on a solid cellulose surface remained stable and active. In addition, the CBM fusions increased the activity of GOOX on soluble glucomannan by up to 30 % and on insoluble crystalline as well as amorphous cellulose by over 50 %.

## Introduction

Oxidation of hydroxyl groups to carbonyls can enhance the gelation, thickening, and metal sequestration potential of polysaccharides [1] and be harnessed to modify corresponding surface properties through subsequent chemical derivatization [2]. Pursuant to these objectives, chemicals such as 2,2,6,6-tetramethylpiperidine-1-oxyl (TEMPO), sodium periodate, and halide ions including I^-^ and Br^-^ have been used to respectively oxidize polysaccharides at primary hydroxyl positions, hydroxyl groups at positions C2 and C3, and hydroxyl groups at positions C1 and C6 [1].

Alternatively, carbohydrate oxidases can facilitate regio-selective oxidation of sugars and polysaccharides, and were recently categorized as auxiliary activities (AA) in the carbohydrate-active enzyme database (http://www.cazy.org) [3]. Examples include, pyranose oxidases (EC 1.1.3.10, AA3_4) [4], glucose 1-oxidases (EC 1.1.3.4, AA3_2) [5], cellobiose dehydrogenase (CDH, EC 1.1.99.18, AA3_1)[6], and galactose 6-oxidase (EC 1.1.3.9, AA5_2) [7, 8].

Whereas the biochemistry and production of the above mentioned oxidases have been studied in detail, oligosaccharide oxidases from family AA7 are comparatively less well characterized. The corresponding carbohydrate oxidases target the C1 hydroxyl of a broad range of oligosaccharides, including cello-, -xylo-, and malto-oligosaccharides. Examples of characterized AA7 enzymes include a gluco-oligosaccharide oxidase (EC 1.1.3) from *Sarocladium strictum* T1 (GOOX-T1) [9], a gluco-oligosaccharide oxidase from *Sarocladium strictum* CBS 346.70 (GOOX) [10], a carbohydrate oxidase from *Microdochium nivale* (MnCO) [11], and a chito-oligosaccharide oxidase from *Fusarium graminearum* (ChitO) [12]. These flavoenzymes are likely to share a conserved FAD-binding domain while showing differences in their substrate binding domains.

In particular, GOOX-T1 and GOOX were previously shown to function best at 37°C and pH 8, and remain stable up to 50°C and 45°C, respectively [10, 13, 14]. Both enzymes oxidize maltose, lactose and cello-oligosaccharides [9, 10]; GOOX was later shown to also oxidize xylo-oligosaccharides and xylan [15, 16]. Given the precedence for GOOX activity on oligomers of glucose, the aim of the current study was to evaluate the potential of selected carbohydrate binding modules (CBMs) to increase GOOX activity on plant polysaccharides, particularly β-glucans.

To date, CBMs have been classified into 71 families based on amino acid sequence similarities (www.cazy.org, 2015). These modules have been further grouped into three types based on folding and substrate specificity [17, 18]. Briefly, Type A CBMs possess a comparatively flat binding site that can associate with crystalline polysaccharides, whereas Type B CBMs adopt a carbohydrate binding groove that binds single glycan chains, and Type C CBMs bind short oligosaccharides.

The varied contributions and significance of CBMs on cellulolytic enzymes was recently reviewed [19]. Whereas certain CBMs can increase non-productive binding to lignin present in lignocellulose [19, 20], CBMs can also improve the performance of cellulolytic enzymes on insoluble substrates, particularly when present at low substrate concentrations [21, 22]. Moreover, CBMs from thermophiles can increase the thermostability of carbohydrate-active enzymes [23, 24]. In a recent study, Telke et al. (2012) found that fusing CBM3, CBM4 or CBM30 to a family GH9 endoglucanase increased enzyme activity on different cellulose preparations by approximately 10-fold [25]. Similarly, Voutilainen et al. (2014) attached various cellulose binding modules from both bacterial and fungal origins to a fungal family GH7 cellobiohydrolase. In all cases, the CBM addition increased enzyme binding to cellulose as well as thermostability, where the addition of a bacterial CBM3 from *Clostridium thermocellum* (CipA) led to highest activity gains [26]. In addition to affecting cellulolytic activity, CBMs can enhance hemicellulase activity, particularly on immobilized and insoluble substrates [27–29]. The impact of CBM fusion to carbohydrate oxidases was also recently investigated. Specifically, a xylan-binding module from family CBM22 was appended to the N terminus of GOOX, which enhanced functional immobilization of the enzyme to xylan coated surfaces [16].

In this study, we evaluated the potential of selected CBMs to increase the binding capacity and activity of GOOX towards insoluble celluloses and β-glucans presented at low concentrations. Specifically, three characterized CBMs were fused to either the N-terminus or C-terminus of GOOX, namely 1) the Type-A *Ct*CBM3 from *C*. *thermocellum* CipA, which can bind cellulose [28], 2) the Type-B *Ct*CBM11 from *C*. *thermocellum* Lic26A-Cel5E, which can bind β-glucan [30], and 3) the Type-B *Ct*CBM44 from *C*. *thermocellum* Cel9D-Cel44A, which can bind xyloglucan, glucomannan and β-glucan [31, 32]. These CBMs were chosen for this analysis since corresponding binding affinities have been characterized; being sourced from a thermophilic organism also presented the possibility to confer thermostability to GOOX as previously observed for cellulolytic enzymes [25, 26]. In addition to directly comparing the impacts of different CBM families as well as N-terminal versus C-terminal fusion on GOOX activity, to our knowledge this work is among the first studies to examine the impact of CBMs from family 11 and 44 on the activity of engineered enzymes.

## Materials and Methods

### Materials

All chemicals were reagent grade, and purchased from Sigma (Canada) unless otherwise specified. Cellobiose was purchased from BioShop Inc. (Canada). Cello-oligosaccharides, barley β-glucan, konjac glucomannan, xyloglucan from tamarind seed, carboxymethyl cellulose, and hydroxyethyl cellulose were purchased from Megazyme (Ireland). Nanocrystalline cellulose was a kind gift from Dr. Y. Boluk (University of Alberta). Regenerated amorphous cellulose was produced from Sigmacell cellulose Type 20 after the cellulose was wetted with water, dissolved in phosphoric acid, and regenerated in water as described previously [33]. The CBM encoding genes from *Clostridium thermocellum*, *Ct*CBM3, *Ct*CBM11, and *Ct*CBM44 were purchased from Nzytech (Portugal). The same genes were codon optimized for expression in *Pichia pastoris* and were synthesized by DNA 2.0 (USA).

### Construction of fusion enzymes

**Fig 1:**
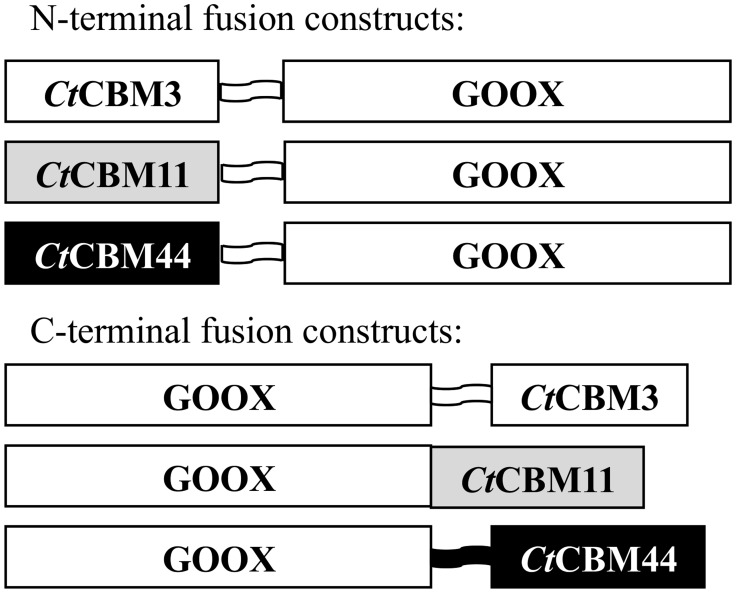
Schematic representation of wild-type GOOX and GOOX fusions. Schematic representation of carbohydrate binding modules fused to the amino and carboxyl terminal end of glucooligosaccharide oxidase from *Sarocladium strictum*. Natural linkers were used in C-terminal fusions while TP linkers were used in N-terminal fusions.

To construct C-terminal CBM fusion proteins, genes encoding *Ct*CBM3, *Ct*CBM11 and *Ct*CBM44 with native N-terminal linkers (*Ct*CBM3: PTNTPTNTPTNTP, *Ct*CBM44: PPPY) or no linker sequence (*Ct*CBM11) were separately cloned into the *Xba*I restriction site of pPICZαA-GOOX, a plasmid for recombinant expression of GOOX-VN in *P*. *pastoris* [10]. Three genes were synthesized by DNA 2.0 to produce corresponding N-terminal CBM fusion proteins. In this case, a DNA sequence encoding a TP linker (SRGGGTATPTPTPTPTP) was inserted between genes encoding *Ct*CBM3, *Ct*CBM11 or *Ct*CBM44, and the gene encoding GOOX (Fig 1). All plasmid constructs were sequenced at the Center for Applied Genomics (the Hospital for Sick Children, Toronto, Canada) before being transferred to *P*. *pastoris* for recombinant protein production.

### Recombinant expression of fusion proteins in *Pichia pastoris*

All plasmids were transformed into *P*. *pastoris* GS115 according to the manufacturer's instructions (Invitrogen, Pichia Expression version G). *P*. *pastoris* transformants were selected on buffered minimal methanol medium containing histidine (BMMH, 100 mM potassium phosphate pH 6.0; 1.34% yeast nitrogen base without amino acids (YNB); 4 x 10^-5^% biotin; 0.5% methanol, 0.004% histidine), and then screened for protein expression using an overlay activity assay. Briefly, the assay mixture (0.3% agarose, 2% cellobiose, 50 mM Tris-HCl pH 8.0, 2 mM phenol, 0.4 mM 4-aminoantipyrine (4-AA), and 15 U/mL horseradish peroxidase) was maintained in a 40°C water bath, and 10 mL of the solution was gently poured on top of each BMMH plate containing the transformant colonies. After solidifying at room temperature for 15 min, plates were transferred to 37°C for 60 min to induce the chromogenic reaction between 4-AA and hydrogen peroxide. Transformants with highest activity were then selected for liquid cultivation.

Selected *P*. *pastoris* transformants were grown overnight in 100 mL of buffered minimal glycerol medium (BMGY, 1% yeast extract; 2% peptone; 100 mM potassium phosphate pH 6.0; 1.34% YNB; 4×10^−5^% biotin; 1% glycerol) at 30°C with continuous shaking at 250 rpm. The cells were harvested by centrifugation at 1,500 × g for 10 min and suspended in 200 mL of BMMH medium supplemented with 1% casamino acid in 1-L flasks to OD_600_ ∼2. Cultures were grown at 27°C and 250 rpm for 4 days and 0.5% methanol was added every 24 h to induce recombinant protein expression. To minimize proteolysis of the secreted recombinant protein, 2 μM leupeptin was added to the culture medium every 24 h.

### Purification of recombinant enzymes

Culture supernatants containing the recombinant protein were harvested by centrifugation at 6,000 × g for 10 min and filtered through 0.22 μm PES filter membrane (GE water and process technologies, USA). The culture supernatants were concentrated and buffer exchanged into binding buffer (100 mM potassium phosphate pH 8, 300 mM NaCl, and 5 mM imidazole) using a Jumbosep centrifugal device (Pall Corp, USA). Resulting concentrates were incubated separately with Ni-NTA resin (Qiagen, Germany) and eluted with 250 mM imidazole; the protein solution was exchanged to 40 mM Tris-HCl pH 8 using Vivaspin 20 concentration units (GE healthcare, UK). Protein concentrations were measured using the Pierce BCA assay (Thermo Scientific, Canada) and confirmed using SDS-PAGE densitometry, where the band density of purified protein and a dilution series of bovine serum albumin (BSA) were determined using ImageJ (http://rsbweb.nih.gov/ij/)[34].

### Specific activity on oligosaccharides, soluble polysaccharides and insoluble cellulose substrates

A chromogenic assay was used to detect and measure hydrogen peroxide production [14]. To measure activity on oligosaccharides, reactions contained 3 pmol of enzyme, 0.1 mM 4-aminoantipyrine, 1 mM phenol, 0.5 U horseradish peroxidase, 50 mM Tris—HCl (pH 8.0), and 0.1 mM of each substrate; 20 mM CaCl_2_ was also included in reaction mixtures as *Ct*CBMs from family 3, 11 and 44 show conserved calcium binding sites [31, 35, 36]. Reactions without enzyme served as negative controls. In all cases, the final reaction volume was 250 μL. The production of hydrogen peroxide was coupled to the reduction of 4-aminoantipyrine by horseradish peroxidase and detected at 500 nm. One unit of the GOOX activity corresponded to the formation of 1 μmol of the product per min. Reactions were incubated at 37°C for 10 min and absorbance was measured every 1 min. Hydrogen peroxide (0–0.04 mM) was used to generate a standard curve. All experiments were performed as triplicates.

To determine kinetic parameters on cellotetraose, initial rates of reactions were measured using the above assay with eight substrates concentrations from 0.01–1 mM. Kinetic parameters were then calculated using the Michaelis—Menten equation (GraphPad Prism5 Software).

The standard reaction described above was also used to measure enzyme activity on soluble polysaccharides. Since GOOX activity significantly differed on each test polysaccharide, enzyme and substrate concentrations were adjusted so that activity data could be collected within the linear range of the standard curve for the assay. Accordingly, between 7.7 pmol and 30 pmol of enzyme was added to the assay mixture, and reactions contained 0.1% to 0.5% (w/v) of test polysaccharides. Specifically, reactions containing 7.7 pmol of enzyme were with 0.5% xyloglucan, whereas 30 pmol of enzyme were used with 0.1% konjac glucomannan, 0.3% barley β-glucan, 0.3% carboxymethyl cellulose, and 0.5% hydroxyethyl cellulose. All reactions were incubated at 37°C for 30 min and the absorbance was measured every 5 min.

Similarly, to measure enzyme activity on insoluble substrates, between 7.7 pmol and 30 pmol of enzyme was added to the standard assay mixture, and reactions contained 0.2% and 0.5% of test polysaccharides. Specifically, reactions containing 7.7 pmol of enzyme were with 0.5% of microcrystalline cellulose (Avicel pH-101), 0.5% of nanocrystalline cellulose, and 0.5% of oat spelt xylan, whereas 30 pmol of enzyme were used with 0.2% of regenerated amorphous cellulose. All reactions were incubated with mixing (500 rpm) at 37°C for up to 24 h using an Eppendorf Thermomixer equipped with an adaptor for 96-well plates. To avoid evaporation, microplates were sealed using an adhesive sheet. For each time point (0, 2, 4, 6, and 24 h) the entire 250 μL reaction was collected, centrifuged to precipitate the insoluble fraction, and then 150 μL of the supernatant was used to read absorbance at 500 nm.

### Cellulose binding

The binding of wild-type GOOX and CBM fusions to microcrystalline cellulose (Avicel pH-101) and regenerated amorphous cellulose was determined by mixing 10 μg of enzyme with 0.5 mg of the cellulose sample in 250 μL of a buffer solution (20 mM CaCl_2_, 0.05% Tween 20, 50 mM Tris-HCl pH 8). Following incubation for 2 h at 4°C with vigorous shaking at 1,400 rpm, mixtures were centrifuged to recover supernatants containing the unbound protein fraction and pellets containing the bound protein fraction. Supernatant samples were concentrated to 20 μL by vacuum centrifugation while pellets were washed three times with the buffer solution before being extracted for 10 min at 100°C with 20 μL of a denaturing solution (10% SDS and 10% β-mercaptoethanol). The bound and unbound fractions were then analyzed by SDS-PAGE and the proteins were visualized by Coomassie blue staining.

### Quartz crystal microbalance with dissipation (QCM-D)

QCM-D experiments were performed with cellulose-coated sensors (QSX 334, Q-Sense, Sweden) using the Q-Sense E4 instrument (Q-Sense, Sweden). Briefly, this instrument detects adsorption of materials to the sensor surface by measuring changes in oscillation frequency and dissipation values dictated by the mass and viscosity of the bound material. The flow rate was kept constant at 0.05 mL/min and the temperature was maintained at 25°C. The changes in areal mass (ng/cm^2^) were obtained using the Voigt model of the Q-Tools software (Q-sense, Sweden). All enzyme and substrate solutions were prepared in a reaction buffer of 50 mM Tris-HCl pH 8.0. The sensors were equilibrated with 50 mM Tris-HCl pH 8.0 for approximately 16 h, and then 1.5 μg/mL of *Ct*CBM3-GOOX or the wild-type GOOX was flowed over the coated sensors until the frequency and dissipation values stabilized. The protein solutions were then replaced by the equilibration buffer to rinse away unbound materials, and after washing, the equilibration buffer was replaced by 0.5 mM cellobiose and the flow-through was collected. Following 40 min of reaction between bound GOOX and cellobiose, corresponding quartz sensors were removed from the QCM-D and washed 3 times with the equilibration buffer before being incubated for 24 h with 800 μL of 0.5 mM cellobiose. In this way, reaction products were allowed to accumulate in the reaction mixture, which facilitated product detection. The cycle wash and batch incubation of GOOX-immobilized sensors with 0.5 mM cellobiose was repeated 3 times. The presence of hydrogen peroxide in the flow-through from the QCM-D, as well as in the sensor incubation solution, was detected using the standard chromogenic assay.

### Affinity gel electrophoresis

Binding of GOOX and fusion constructs to konjac glucomannan, barley β-glucan, xyloglucan from tamarind seed, and carboxymethyl cellulose was examined by native affinity gel electrophoresis as described by Freelove et al. (2001) [37] with minor modifications. Briefly, the native polyacrylamide gels prepared for these analyses contained 7.5% (w/v) acrylamide in 25 mM Tris, 250 mM glycine buffer (pH 8.3), and 0.01% of the test polysaccharide. Approximately 5 μg of GOOX and each fusion construct were loaded onto the gels and then run at 90 V for 2 h at room temperature. Relative binding affinities were inferred from the migration distance of the fusion proteins and wild-type GOOX on gels with and without the test polysaccharides. BSA (5 μg) was also used as a reference protein for these analyses.

### Temperature stability

To investigate the potential of each CBM to increase the temperature stability of GOOX, 0.5 μg of each fusion protein or wild-type GOOX was incubated for up to 4 h at 45°C, before being cooled to room temperature to measure residual enzyme activity using the GOOX standard assay and 1 mM cellobiose as substrate. All experiments were performed in triplicate. The half-life was measured by plotting the logarithm of percent remaining activity versus the incubation time using Microsoft Excel (v 14.1.4).

### Nucleotide sequence accession number

The genes encoding N-terminal *Ct*CBM3-GOOX, *Ct*CBM11-GOOX, *Ct*CBM44-GOOX and C-terminal GOOX-*Ct*CBM3, GOOX-*Ct*CBM11, and GOOX-*Ct*CBM44 have been deposited in the GenBank database under accession numbers: JX181765, JX181766, JX181767, JX181768, JX181769, and JX181770, respectively.

## Results and Discussion

### Recombinant protein production

When using the standard *Pichia* expression protocol to produce N-terminal CBM fusions, degradation products that corresponded to the size of the wild type GOOX and CBMs separately were observed by SDS-PAGE. This observation implied proteolysis of the N-terminal fusion proteins at the linker site. *P*. *pastoris* is known to release extracellular serine, cysteine and asparatic proteases [38]. The activity of these proteases depends on the pH, where serine protease activity is highest above pH 7, cysteine-type protease activity is highest between pH 5–7, and aspartic protease activity is highest below pH 5 [38]. In cases where proteolysis of recombinant proteins is observed, optimization of expression parameters can significantly improve protein expression. For example, lowering the induction temperature can reduce cell lysis and thereby minimize the release of extracellular proteases; casamino acids can compete with the recombinant protein for extracellular protease action and thereby protect the expressed protein from degradation [38]; daily addition of protease inhibitors can also increase protein expression in *P*. *pastoris* [39]. Accordingly, in the present work the above parameters were evaluated as a means of improving the functional, recombinant expression of intact GOOX fusions.

Lowering the induction temperature to 15°C led to protein aggregation. However, slight reduction of temperature to 27°C along with supplementation of 1% casamino acid and daily addition of 2 μM leupeptin, significantly improved the expression of soluble, intact fusion proteins. When using the improved cultivation conditions, the yield of the C- and N- terminal constructs were similar, suggesting that the codon optimization used for N-terminal constructs did not significantly increase recombinant protein expression in *P*. *pastoris*. Moreover, the susceptibility of N-terminal fusion proteins to proteolysis at the linker site suggests that in this case, the natural linkers used in the C-terminal constructs were more resistant than the synthetic TP linker to secreted proteases.

The average yield of fusion and wild-type enzymes was approximately 5 mg/L and the Ni-NTA purification system recovered wild-type and fusion proteins to over 95% purity, as judged by SDS-PAGE (S1 Fig). The activities of the fusion proteins on cellobiose were comparable or higher than that of wild-type GOOX with the exception of GOOX-*Ct*CBM3, which was approximately 80% of the wild-type activity (Table 1).

**Table 1:** Activity (U/ μmol) of wild-type GOOX and CBM fusions on 0.1 mM cello-oligosaccharides.

Enzymes	Cellobiose	Cellotetraose	Cellohexaose
Wild-type GOOX	244 ± 7	197 ± 8	150 ± 2
*Ct*CBM3-GOOX	304 ± 4	253 ± 7	194 ± 3
*Ct*CBM11-GOOX	287 ± 1	235 ± 6	178 ± 6
*Ct*CBM44-GOOX	325 ± 6	256 ± 12	201 ± 4
GOOX-*Ct*CBM3	200 ± 8	166 ± 12	126 ± 0
GOOX-*Ct*CBM11	344 ± 24	279 ± 12	213 ± 4
GOOX-*Ct*CBM44	291 ± 3	239 ± 11	183 ± 4

All CBM fusions led to statistically significant differences in specific activities compared to wild-type GOOX as determined using a two-tailed t-test for two samples with unequal variance (p < 0.05). Errors indicate standard deviations; n = 3.

### Improved binding to polymeric substrates

**Fig 2:**
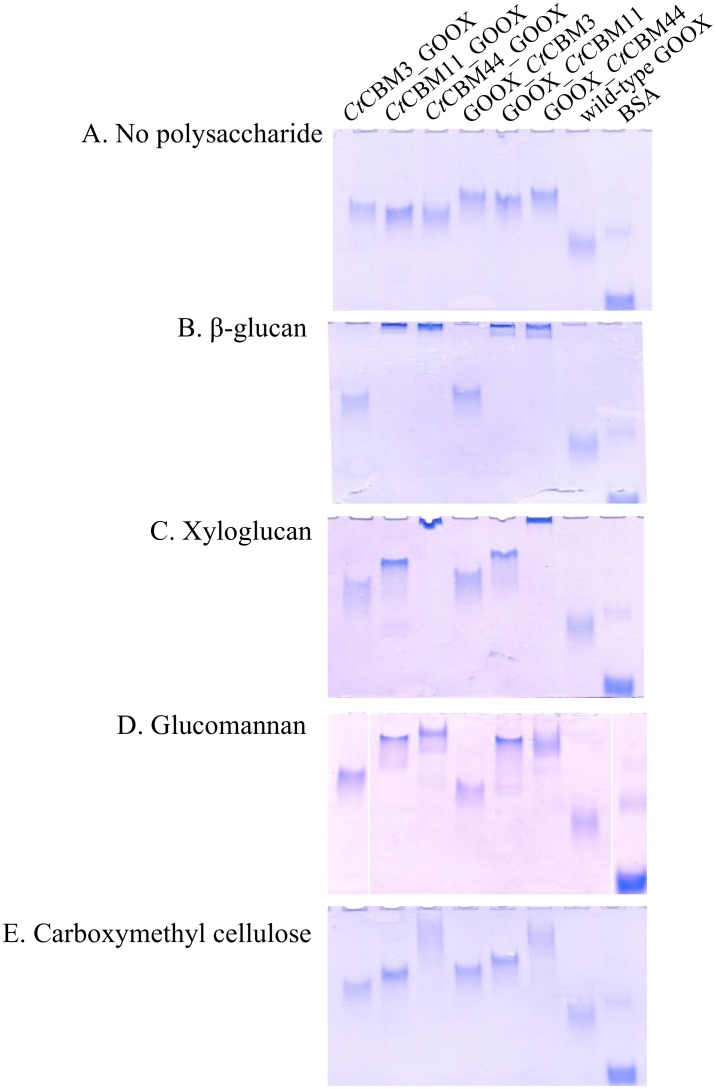
Affinity gel electrophoresis (AGE) of wild-type GOOX and CBM fusions. Purified proteins were subjected to AGE using a 7.5% (w/v) polyacrylamide gel containing A: no polysaccharides, B: β-glucan, C: xyloglucan, D: glucomannan, E: carboxymethyl cellulose. The final concentration of each polysaccharide was 0.01%. Bovine serum albumin (BSA) was used as a reference.

GOOX fusion to selected CBMs significantly improved GOOX binding to tested polysaccharides, and binding selectivity was observed according to the appended CBM. For instance, *Ct*CBM3 fusion increased GOOX binding to crystalline cellulose (Avicel) and regenerated amorphous cellulose by more than 10-fold (Table 2, S2 Fig). As expected, fusion to *Ct*CBM11 and *Ct*CBM44 increased the affinity of GOOX towards soluble polysaccharides, including β-glucan, glucomannan, and xyloglucan (Fig 2); fusion of GOOX to *Ct*CBM11 or *Ct*CBM44 also promoted enzyme binding to regenerated amorphous cellulose (Table 2, S2 Fig). Overall, binding results were consistent with previous studies that confirmed *Ct*CBM11 and *Ct*CBM44 affinity towards β-glucan, lichenan, hydroxyethyl cellulose, glucomannan and oat spelt xylan, along with *Ct*CBM44 binding to xyloglucan [30, 31, 36]. However, to our knowledge, binding of *Ct*CBM11 and *Ct*CBM44 to regenerated amorphous cellulose has not been previously reported.

**Table 2:** Binding of wild-type GOOX and CBM fusions to insoluble cellulose.

Enzyme	Portion bound (%)	
	Avicel	RAC
Wild-type GOOX	6	7
*Ct*CBM3-GOOX	60	94
*Ct*CBM11-GOOX	4	65
*Ct*CBM44-GOOX	6	74
GOOX-*Ct*CBM3	53	98
GOOX-*Ct*CBM11	3	67
GOOX-*Ct*CBM44	6	95

The bound and unbound fractions were analyzed by SDS-PAGE (S2 Fig), and the percentage of bound protein was calculated by measuring protein band density using ImageJ.

In most cases, binding was not affected by CBM positioning at the N-terminus or C-terminus of GOOX (Fig 2, Table 2). The exception was *Ct*CBM44 fusions, where C-terminal constructs showed slightly better binding to regenerated amorphous cellulose than corresponding N-terminal constructs. Notably, the N-terminus of *Ct*CBM44 contains a PKD domain, which would extend the flexible linker region when *Ct*CBM44 is fused to the C-terminus of GOOX.

### Activity on oligosaccharides

With the exception of C-terminal *Ct*CBM3 fusion, all CBM fusions generated herein increased GOOX activity on 0.1 mM cellobiose, cellotetraose and cellohexaose (Table 1). Similar results were previously reported for GOOX activity on cello-oligosaccharides and xylo-oligosaccharides after fusion to xylan-binding *Ct*CBM22 [16]. Subsequent kinetic analyses performed herein revealed that increased activities were best explained by slight but statistically significant increases in *k*_cat_ for all N-terminal fusions as well as the C-terminal *Ct*CBM11 construct (Table 3). Likewise, the reduced activity of GOOX-*Ct*CBM3 on all cellooligosaccharides could be explained by a decrease in *k*_cat_.

**Table 3:** Kinetics parameters of wild-type GOOX and CBM fusions on cellotetraose.

Enzymes	*k*_cat_ (min^-1^)	*K*_m_ (mM)	*k*_cat_/*K*_m_ ^a^ (min^-1^. mM^-1^)
Wild-type GOOX	250 ± 20 ^a^	0.07 ± 0.02	3,400 ± 700
*Ct*CBM3-GOOX	300 ± 20*	0.10 ± 0.02	3,100 ± 700
*Ct*CBM11-GOOX	310 ± 20*	0.10 ± 0.02	3,100 ± 700
*Ct*CBM44-GOOX	380 ± 30*	0.12 ± 0.03	3,100 ± 700
GOOX-*Ct*CBM3	150 ± 10	0.05 ± 0.01	2,900 ± 600
GOOX-*Ct*CBM11	360 ± 30*	0.11 ± 0.02	3,200 ± 700
GOOX-*Ct*CBM44	290 ± 20	0.09 ± 0.02	3,300 ± 700

Errors indicate standard deviations; n = 3.

^a^ Standard deviations (SD) for *k*_cat_/*K*_m_ values were calculated using following formula: SD *k*_cat_/*K*_m_ = *k*_cat_/*K*_m_ × [SQRT((SD(*K*_m_)/*K*_m_)^2^ + (SD(*k*_cat_)/*k*_cat_)^2^)].

Asterisk indicates statistically significant increases compared to wild-type GOOX as determined using a two-tailed t-test for two samples with unequal variance (p < 0.05).

The increase in *k*_cat_ values on oligosaccharides observed for the N-terminal fusions constructed herein was even greater in earlier studies where *Ct*CBM22A was fused to the N-terminus of GOOX [16]. In all cases, the same artificial TP linker was encoded between the N-terminal CBM and GOOX. However in the case of the earlier *Ct*CBM22 fusion, the TP linker was extended towards the CBM by a 9-amino acid loop (AVAGTVIEG), whereas the TP linker was extended by only 1 to 3 amino acids in the GOOX fusions generated herein. As previously proposed, CBM addition [16] or mutation [15] at the N-terminus of GOOX could cause a conformational change in the proximal FAD-binding domain, thereby affecting the redox potential of the enzyme. We reasoned that a more flexible linker would likely have a greater impact on the conformation of the FAD-binding domain, which could explain the nearly 2-fold increase in *k*_cat_ values upon *Ct*CBM22 fusion [16] compared with approximately 0.5-fold increase in *k*_cat_ values observed in the current study (Table 3). By contrast, the slight increase in *k*_cat_ values for the C-terminal fusion of *Ct*CBM11 construct was correlated to the specificity of CBMs from family 11 towards short oligosaccharides including cellotetraose [36], which is anticipated to promote functional associations between the substrate and substrate binding site that is positioned at the C-terminal end of GOOX. The decrease in *k*_cat_ observed for GOOX-*Ct*CBM3 is more difficult to rationalize. However, since *k*_cat_ is a function of both the catalytic rate constant and rate constant for dissociation of the product, it is conceivable that product release is reduced upon C-terminal positioning of the cellulose-binding *Ct*CBM3 and corresponding linker sequence.

The addition of *Ct*CBM22A to GOOX did not affect *K*_m_ values for oligosaccharides [16]. Likewise in this study, statistically significant changes in *K*_m_ values were not observed when using cellotetraose, suggesting that the CBMs used herein do not compete with -1 or -2 substrate binding subsites of GOOX [10].

### Specific activity on polymeric substrates

Specific activities were then tested using both soluble and insoluble polysaccharides. Oxidation by wild-type GOOX or GOOX fusions was not detected on nanocrystalline cellulose, total oat spelt xylan, or hydroxyethyl cellulose. However, activity of wild-type GOOX was detected for the first time on konjac glucomannan, barley β-glucan, carboxymethyl cellulose, regenerated amorphous cellulose, xyloglucan from tamarind seed, and Avicel (Table 4). In the case of glucommanan, it is likely that the reducing end glucose is mainly oxidized, given the low activity of GOOX on mannose [10].

**Table 4:** Activity of wild-type GOOX and CBM fusions on soluble and insoluble plant polysaccharides.

Activity (U/mmol)						
	Konjac glucomannan (0.1%)	Regenerated amorphous cellulose (0.2%)	Barley β-gluan (0.3%)	Carboxymethyl cellulose (0.3%)	Tamarind seed xyloglucan (0.5%)	Avicel (0.5%)
Wild-type GOOX	4,100 ± 300	290 ± 30	1,520 ± 90	600 ± 100	118 ± 3	79.2 ± 0.3
*Ct*CBM3-GOOX	4,800 ± 100*	420 ± 10*	1,700 ± 100	630 ± 60	120 ± 20	123± 7*
*Ct*CBM11-GOOX	5,100 ± 100*	430 ± 40*	1,300 ± 200	560 ± 50	118 ± 6	86 ± 4
*Ct*CBM44-GOOX	5,400 ± 200*	410 ± 50*	1,700 ± 200	500 ± 100	112 ± 5	97 ± 8
GOOX-*Ct*CBM3	4,100 ± 300	320 ± 20	1,500 ± 200	620 ± 50	110 ± 10	100 ± 4*
GOOX-*Ct*CBM11	5,000 ± 400*	400 ± 30*	1,500 ± 400	500 ± 90	120 ± 20	84 ± 7
GOOX-*Ct*CBM44	5,400 ± 200*	460 ± 60*	1,600 ± 200	500 ± 200	113 ± 3	99± 4*

Substrate concentrations were optimized to measure initial rates of reaction and are indicated in parentheses; Errors indicate standard deviations; n = 3. Asterisk indicates statistically significant improvements compared to wild-type GOOX as determined using a two-tailed t-test for two samples with unequal variance (p < 0.05).

**Fig 3:**
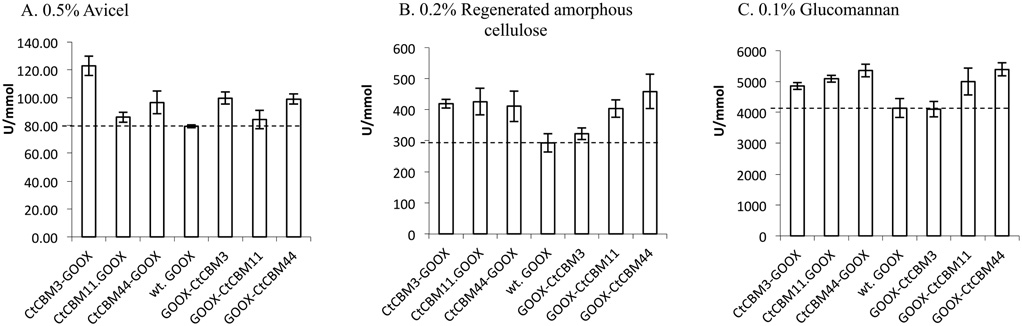
Activity of wild-type GOOX and CBM fusions on polysaccharides. A: crystalline cellulose (Avicel, 0.5%), B: regenerated amorphous cellulose (RAC, 0.2%), and C: glucomannan from konjac (0.1%). Substrate concentrations were optimized to measure initial rates of reaction. One unit corresponds to 1 μmol of product per min. Error bars represents standard deviations; n = 3. The doted line represents the activity of wild-type GOOX.

In experiments using polysaccharides, substrate concentrations were optimized to measure initial rates of reaction. While different substrate concentrations complicate comparisons between substrates, this analysis permitted comparisons relative to wild-type GOOX for a given substrate, which was the main objective of the study. Relative to wild-type GOOX, CBM fusions led to statistically significant improvements in GOOX activity on 0.5% Avicel, 0.2% regenerated amorphous cellulose, and 0.1% konjac glucomannan (Fig 3). For example, *Ct*CBM3 and *Ct*CBM44 fusions increased GOOX activity on Avicel by up to 56%, where the highest enhancement was observed with the N-terminal *Ct*CBM3 fusion. Consistent with this trend, Telke et al. (2012) and Voutilainen et al. (2014) also report greatest enhancement of activity on Avicel upon fusion of *Ct*CBM3 to an endoglucanase from *Alicyclobacillus acidocaldrious* (Cel9A) [25], and a cellobiohydrolase from *Talaromyces emersonii* (Cel7A)[26]. Comparable to specific activities reported for oligosaccharides (Table 1), all CBM fusions with the exception of GOOX-*Ct*CBM3, increased GOOX activity on regenerated amorphous cellulose and glucomannan by up to 55% and 30%, (Fig 3).

The improvement to GOOX activity was comparable to previous studies reporting 20–50% increased performance of a cellodextrin phosphorylase on regenerated amorphous cellulose upon addition of a CBM9 [40], and the 10–80% improved activity of Cel9A *Alicyclobacillus acidocaldrious* on β-glucan upon fusion of a CBMs from families 3,4 and 30 [25]. Highest improvement was observed herein with fusion to *Ct*CBM44, where impacts were approximately two times higher when using 0.1% glucomannan compared to 0.3% (S3 Fig). This result, along with highest activity improvement on Avicel and regenerated amorphous cellulose, is consistent with previous reports showing greatest impact of CBMs on glycoside hydrolase activity when using insoluble substrates or relatively low concentrations of soluble polysaccharides [22].

Changes in activity values were generally correlated the selectivity of the respective CBM. For instance, the increased activity of *Ct*CBM3-GOOX on Avicel compared to wild-type GOOX was consistent with improved binding of this fusion protein to the substrate (Table 2), as well as the previously reported affinity of *Ct*CBM3 towards microcrystalline cellulose [28]. Similarly, the highest activity of GOOX-*Ct*CBM44 on glucomannan was correlated to the relatively high binding of this construct on glucomannan (Fig 2D) and the previously reported affinity of *Ct*CBM44 towards this polysaccharide [31].

Even though CBM fusion improved GOOX binding to all tested polysaccharides, it did not increase GOOX oxidation of barley β-glucan, carboxymethyl cellulose or xyloglucan. Whereas barley β-glucan comprises mixed β-(1→3)- and β-(1→4)-linkages and is unbranched, xyloglucan and carboxymethyl cellulose have β-(1→4)-linked glucose backbones that are substituted with branching sugars or carboxymethyl groups, respectively. An early study of gluco-oligosaccharide oxidases did not detect GOOX activity on β-(1→3)-linked glucose of laminaribiose [41], and a more recent study showed reduced GOOX activity on branched xylo-oligosaccharides and anionic xylo-oligosaccharides [15]. It is therefore likely that the mixed-linkage backbone structure of barley β-glucan, and branching groups in carboxymethyl cellulose and xyloglucan, restrict functional interactions between these substrates and the -1 and -2 subsites of GOOX [15, 42].

### Immobilization of GOOX through *Ct*CBM3

In addition to enhancing activity on cellulose, we postulated that *Ct*CBM3 fusion could promote GOOX immobilization to cellulosic surfaces, which is relevant to several applications including the use of enzymes in biosensing materials. Given the comparatively high activity of *Ct*CBM3-GOOX, additional comparative analyses were restricted to *Ct*CBM3-GOOX and wild-type GOOX, this time using a quartz crystal microbalance with dissipation (QCM-D) equipped with cellulose-coated piezoelectric crystal sensors.

**Fig 4:**
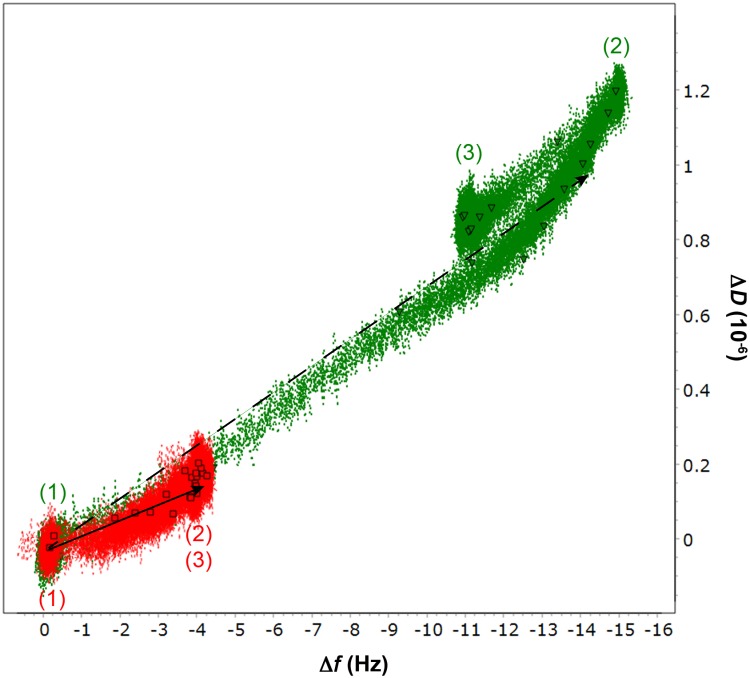
Frequency—dissipation plot of wild-type GOOX and *Ct*CBM3-GOOX binding to cellulose. Changes in frequency (Δ*f*) and dissipation (Δ*D*) during 1.5 μg/mL enzyme addition (1), 50 mM Tris-HCl pH 8 buffer washing (2) and 0.5 mM cellobiose addition (3) in the experiments with *Ct*CBM3-GOOX (green, triangle) and wild-type GOOX (red, square). The total running time is 210 min. Linear fitting for cellulose binding of the CBM fusion (dashed arrow) and the wild-type (solid arrow) were analyzed by GraphPad Prism 5.

In this analysis, protein adsorption was observed as a decrease in oscillation frequency of the sensor, whereas increase in dissipation reflects a more viscoelastic surface layer. Accordingly, the higher change in frequency (Δ*f*) observed using *Ct*CBM3-GOOX confirmed enhanced binding of this enzyme to cellulose compared to the wild type GOOX (Fig 4; S4 Fig). Considering corresponding differences in molecular weight, Δ*f* values corresponded to a molar adsorption ratio of the fusion and wild-type enzymes of approximately 1.6. Notably, the slight increase in slope (ΔD/Δ*f*) of the D-*f* plot depicting *Ct*CBM3-GOOX binding suggests a more viscoelastic surface layer was formed by the fusion enzyme (Fig 4).

After extensive washing to remove loosely bound enzyme, cellobiose was passed over the sensors coated with immobilized *Ct*CBM3-GOOX or GOOX. The addition of cellobiose resulted in negligible changes to frequency and dissipation (Fig 4), indicating that the enzymes remained bound to cellulose in the presence of the soluble substrate. Moreover, the activity of immobilized *Ct*CBM3-GOOX and GOOX was confirmed by measuring H_2_O_2_ after batch incubation of recovered sensors with 0.5 mM cellobiose. Given that measured activities remained stable after repeated cycles of washing and incubation of the sensors with cellobiose (S5 Fig), this analysis demonstrated that fusion of GOOX to *Ct*CBM3 could have advantages beyond increased activity on cellulose, including one-step purification of active enzyme from culture media based on cellulose adsorption, as well as biosensing and biofuel cell applications involving mixed sugars.

### Effect of CBM fusion on thermostability

Varying effects of CBM fusion on the temperature stability of associated enzymes have been reported. For instance, Jun et al. (2009) show that appending a xylan specific CBM from *Thermotoga maritima* to xylanase 2 from *Hypocrea jecorina* improves the thermostability and substrate affinity of the enzyme [43]. In another study, fusion of a family 42 CBM from *Aspergillus kawachii* to a feruloyl esterase from *A*. *awamori* was shown to increase enzyme stability and affinity towards arabinoxylan [44]. However, while fusion of a family 6 CBM from *Clostridium stercorarium* Xy1A to *Bacillus halodurans* Xy1A does not affect enzyme stability [45], fusion of a family 22 CBM to *B*. *halodurans* C-125 family 10 xylanase decreases the thermostability of the enzyme [29]. The range in effects of CBMs on protein stability is further exemplified by Kataeva et al. (2001), who show that the native CBM4 of CelK from *C*. *thermocellum* increases the thermostability of the catalytic module, which is not retained when substituting CBM4 for a CBM6 encoded by the same organism [46].

Earlier studies of wild-type GOOX confirmed its stability at 40°C, but loss in over 30% and 90% activity after 1 h at 45°C and 50°C, respectively [47]. Therefore, to evaluate the impact of CBM fusion on the thermostability of this enzyme, the half life of wild-type GOOX and CBM fusions were compared at 45°C.

Despite selecting CBMs from a thermophilic bacterium, their fusion to GOOX had negligible impact on the temperature stability of corresponding fusion proteins (Table 5, S6 Fig). Still, slight improvements were observed upon C-terminal fusion of *Ct*CBM44 and N-terminal fusion of *Ct*CBM3 and *Ct*CBM11, which increased the half-life of GOOX at 45°C by up to 40%. It is interesting to note that the impact of CBM fusion on GOOX stability was more readily explained by the nature of the linker region rather than CBM family. For example, the rigidity of the short linker and connecting loops discussed earlier might restrict heat-induced conformational shifts in the N-terminal catalytic domain of GOOX, which could explain the relative thermostabilities of N-terminal fusions (Table 5). This notion was further evident when comparing linker sequences of C-terminal fusions. Specifically, the higher half-life of GOOX-*Ct*CBM44 compared to other C-terminal fusions could be explained by the PPPY linker sequence between GOOX and *Ct*CBM44, which likely adopts a more rigid conformation compared to the longer linker of *Ct*CBM3 (PTNTPTNTPTNTP) and the connecting loop of *Ct*CBM11 (SRAVGE). The potential impact of linker sequences on thermostability is interesting in the context of an earlier report by Dias et al. (2004), who showed that the thermostability of *C*. *thermocellum* xylanase Xyn10B was retained after removing the CBM22 domain and leaving the linker sequence, whereas removing the whole linker-CBM22 sequence reduced the thermostability of the enzyme [48]. Moreover, cellulase fusions with either a flexible polyglycine linker or a rigid alpha-helix linker showed that the rigid linker significantly enhanced enzyme activities and thermostability [49].

**Table 5:** The half-life of wild-type GOOX and CBM fusions at 45°C.

Enzymes	Half life (min)
Wild-type GOOX	119 ± 4
*Ct*CBM3-GOOX	132 ± 2*
*Ct*CBM11-GOOX	132 ± 2*
*Ct*CBM44-GOOX	127 ± 4
GOOX-*Ct*CBM3	104 ± 9
GOOX-*Ct*CBM11	111 ± 10
GOOX-*Ct*CBM44	170 ± 20*

Values were obtained by plotting the log (% residual activity) versus incubation time (min). Errors indicate standard deviations; n = 3. Asterisk indicates statistically significant improvements compared to wild-type GOOX as determined using a two-tailed t-test for two samples with unequal variance (p < 0.05)

## Conclusions

A selection of CBMs with affinity towards different β-glucans were appended to either the C-terminus or N-terminus of GOOX, and fusion proteins with similar or higher yield than the wild type enzyme were successfully expressed and purified from *P*. *pastoris*. All N-terminal fusion proteins as well as the C-terminal *Ct*CBM11 fusion showed higher catalytic activity on tested oligosaccharides than wild-type GOOX, suggesting a positive conformational change to the FAD binding domain. In addition, unchanged *K*_m_ values confirmed that the fused CBMs did not compete with the GOOX subsites for oligosaccharide binding. Similar to activity studies, thermostability of fusion constructs was dictated by the nature of the linker sequence rather than CBM type, where more rigid linkers resulted in more stable fusion proteins, underscoring the relevance of linker selection to fusion protein design. Finally, regardless of positioning, CBM fusion promoted GOOX binding to cellulosic and hemicellulosic polysaccharides, and GOOX remained active when immobilized to cellulose through *Ct*CBM3. This result highlights that CBM fusion, in particular the N-terminal *Ct*CBM3, could facilitate applications of GOOX in cellulose-based biosensing devices.

## Supporting Information

S1 Fig Purified of wild-type GOOX and GOOX fusions. Purified wild-type and fusion GOOX proteins on 10% SDS-PAGE. Lane 1: PageRuler protein ladder; 2: *Ct*CBM3-GOOX; 3: *Ct*CBM11-GOOX; 4: *Ct*CBM44-GOOX; 5: wild-type GOOX, 6: GOOX-*Ct*CBM3; 7: GOOX-*Ct*CBM11; 8: GOOX-*Ct*CBM44.
(TIFF)

S2 Fig Binding of wild-type GOOX and CBM fusions to insoluble cellulose as analyzed by SDS-PAGE. Purified proteins were incubated with crystalline cellulose (Avicel) or regenerated amorphous cellulose (RAC) for 2 h on ice with continuous shaking. Unbound (U) and bound (B) protein fractions are shown.
(TIFF)

S3 Fig Activity of wild-type GOOX and CBM fusions on konjac glucomannan A. 0.1%, B. 0.3%. All reactions contained 0.5 μg of enzyme. Error bars represents standard deviations; n = 3. The doted line represents the specific activity of wild-type GOOX.
(TIFF)

S4 Fig Adsorbed mass of wild-type GOOX and *Ct*CBM3-GOOX on cellulose-coated sensors. Changes in adsorbed mass during 1.5 μg/mL enzyme addition (1), 50 mM Tris-HCl pH 8 buffer washing (2) and 0.5 mM cellobiose addition (3) in the experiments with *Ct*CBM3-GOOX (green, solid line) and wild-type GOOX (red, dashed line). Mass values were obtained using the Voigt model.
(TIFF)

S5 Fig Cellobiose oxidation of enzyme-bound sensors. QCM-D sensors that were previously bound with *Ct*CBM3-GOOX were repeatedly washed and incubated with 0.5 mM cellobiose, and the regeneration of oxidized products was measured by the chromogenic assay.
(TIFF)

S6 Fig Thermostability of proteins at 45°C. A: wild-type GOOX, B: *Ct*CBM3-GOOX, C: *Ct*CBM11-GOOX, D: *Ct*CBM44-GOOX, E: GOOX-*Ct*CBM3, F: GOOX-*Ct*CBM11, G: GOOX-*Ct*CBM44.
(TIFF)
