# Supplementary figures and images for: Enhanced Polysaccharide Binding and Activity on Linear β-Glucans through Addition of Carbohydrate-Binding Modules to Either Terminus of a Glucooligosaccharide Oxidase
Source: PLoS One. 2015 May 1;10(5):e0125398. doi: 10.1371/journal.pone.0125398 (PMC4416756; doi:10.1371/journal.pone.0125398)

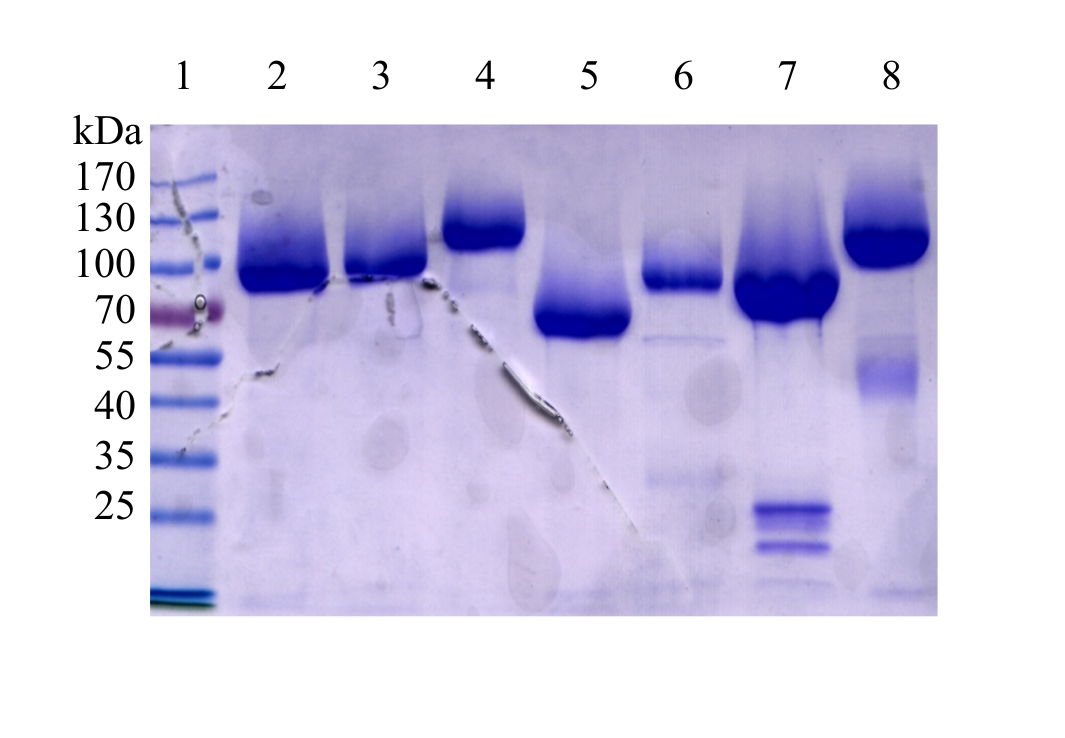

Supplement: S1 Fig — Purified wild-type and fusion GOOX proteins on 10% SDS-PAGE. Lane 1: PageRuler protein ladder; 2: CtCBM3-GOOX; 3: CtCBM11-GOOX; 4: CtCBM44-GOOX; 5: wild-type GOOX, 6: GOOX-CtCBM3; 7: GOOX-CtCBM11; 8: GOOX-CtCBM44. (TIFF) [file pone.0125398.s001.tiff]

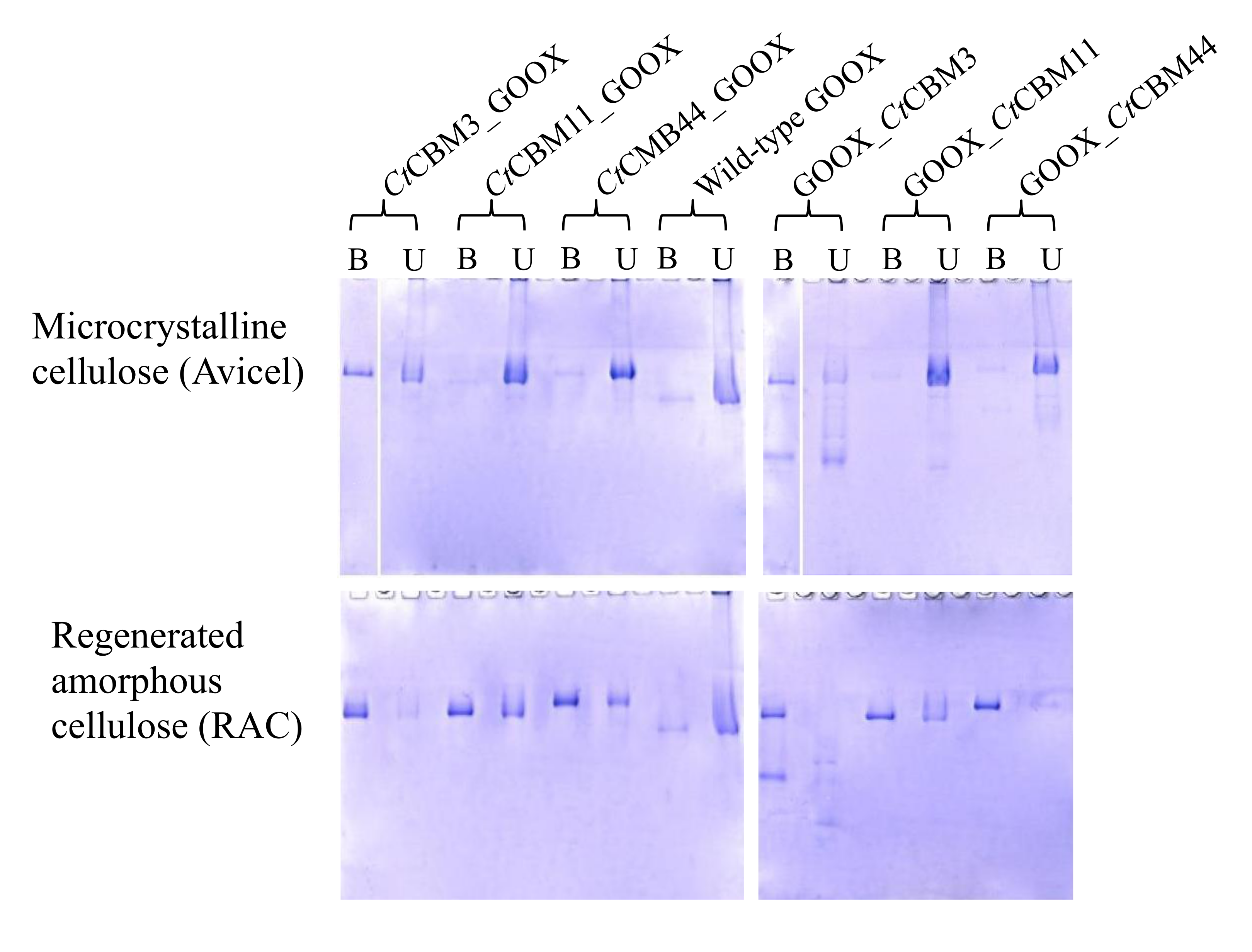

Supplement: S2 Fig — Purified proteins were incubated with crystalline cellulose (Avicel) or regenerated amorphous cellulose (RAC) for 2 h on ice with continuous shaking. Unbound (U) and bound (B) protein fractions are shown. (TIFF) [file pone.0125398.s002.tiff]

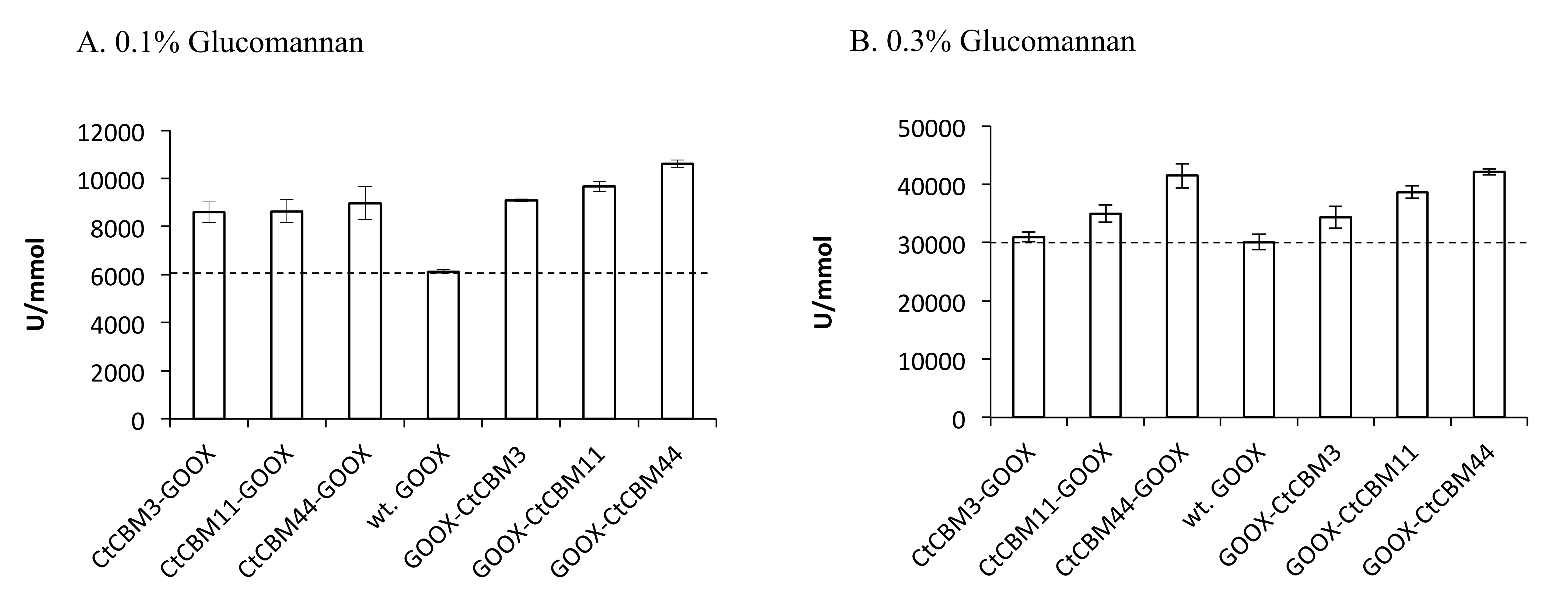

Supplement: S3 Fig — All reactions contained 0.5 μg of enzyme. Error bars represents standard deviations; n = 3. The doted line represents the specific activity of wild-type GOOX. (TIFF) [file pone.0125398.s003.tiff]

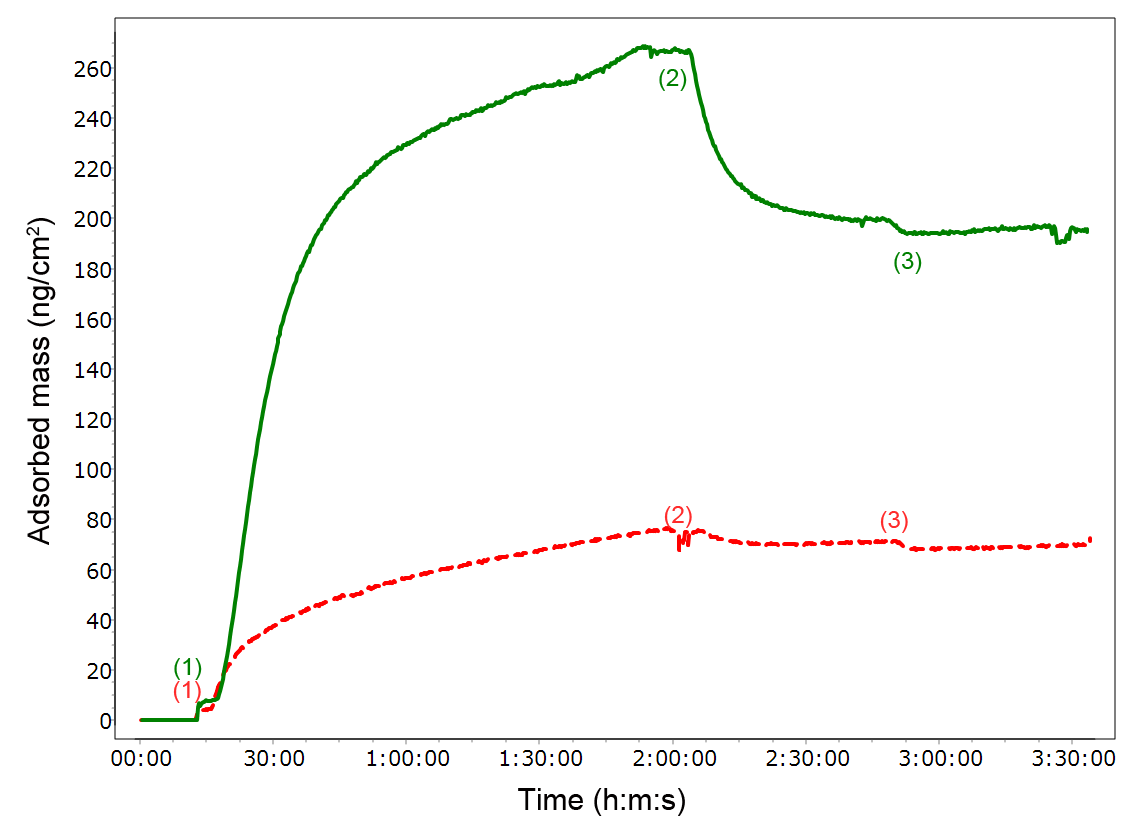

Supplement: S4 Fig — Changes in adsorbed mass during 1.5 μg/mL enzyme addition (1), 50 mM Tris-HCl pH 8 buffer washing (2) and 0.5 mM cellobiose addition (3) in the experiments with CtCBM3-GOOX (green, solid line) and wild-type GOOX (red, dashed line). Mass values were obtained using the Voigt model. (TIFF) [file pone.0125398.s004.tiff]

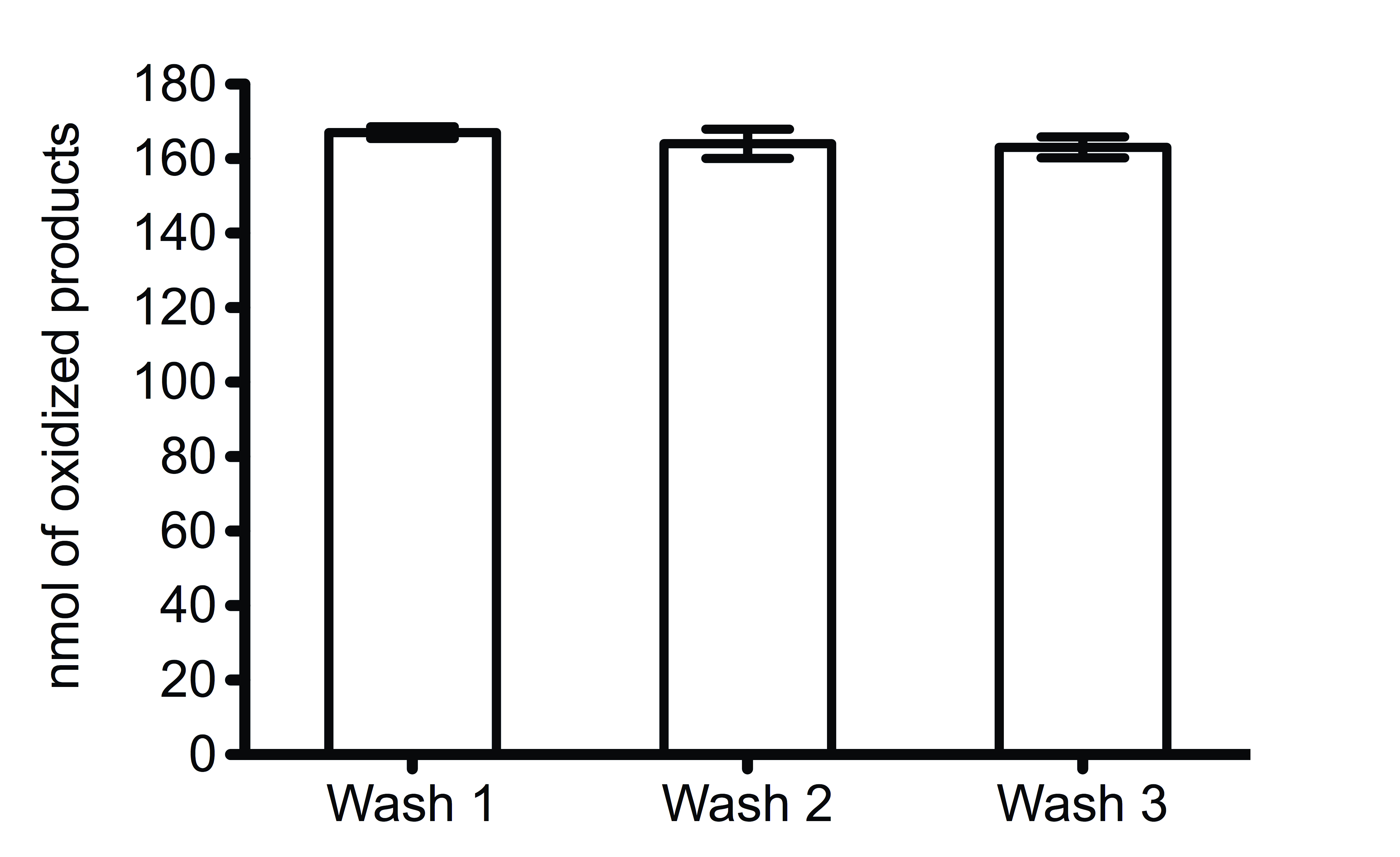

Supplement: S5 Fig — QCM-D sensors that were previously bound with CtCBM3-GOOX were repeatedly washed and incubated with 0.5 mM cellobiose, and the regeneration of oxidized products was measured by the chromogenic assay. (TIFF) [file pone.0125398.s005.tiff]

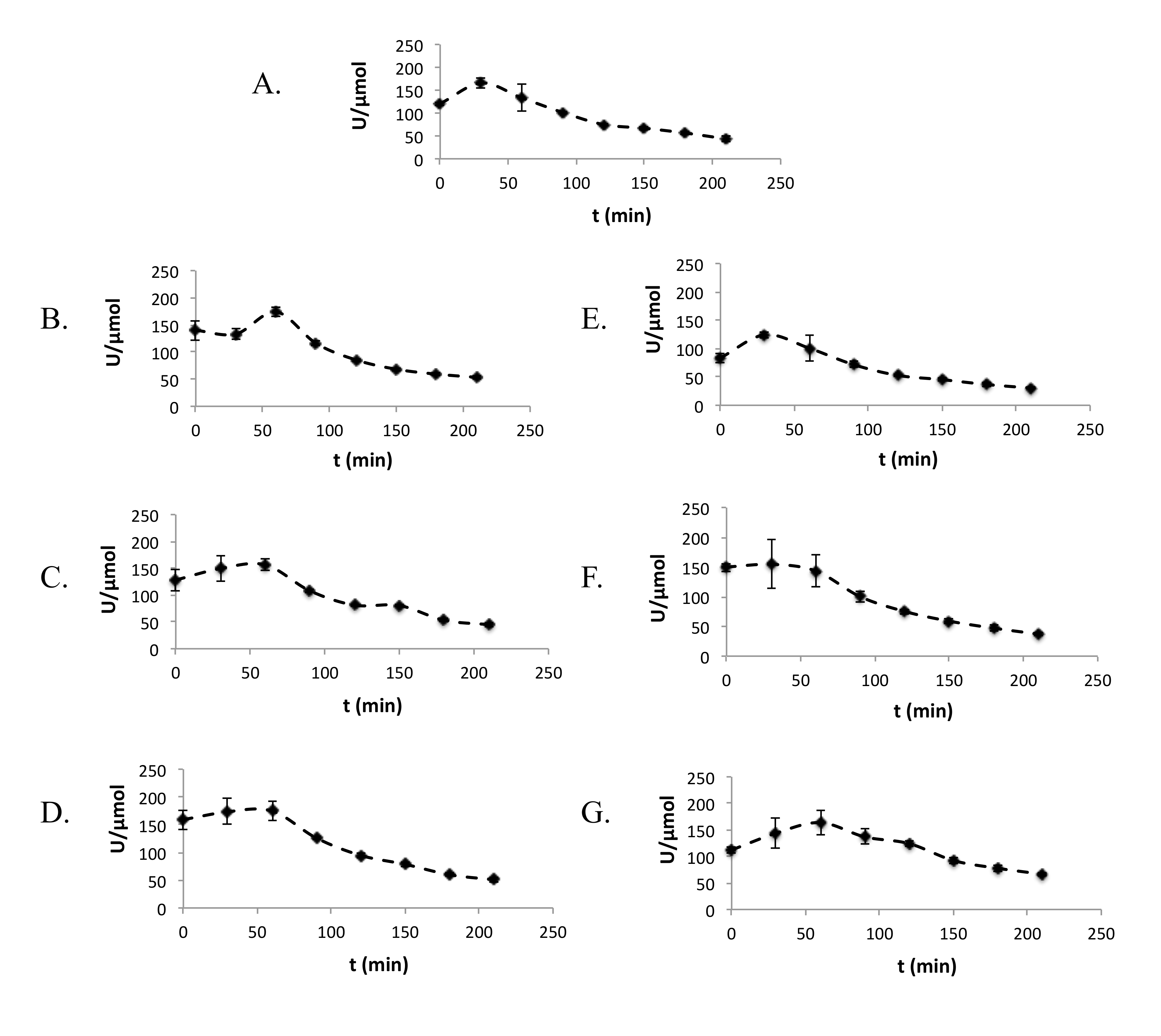

Supplement: S6 Fig — A: wild-type GOOX, B: CtCBM3-GOOX, C: CtCBM11-GOOX, D: CtCBM44-GOOX, E: GOOX-CtCBM3, F: GOOX-CtCBM11, G: GOOX-CtCBM44. (TIFF) [file pone.0125398.s006.tiff]
